# Small-area level socio-economic deprivation and tuberculosis rates in England: An ecological analysis of tuberculosis notifications between 2008 and 2012

**DOI:** 10.1371/journal.pone.0240879

**Published:** 2020-10-19

**Authors:** Patrick Nguipdop-Djomo, Laura C. Rodrigues, Ibrahim Abubakar, Punam Mangtani

**Affiliations:** 1 Department of Infectious Disease Epidemiology, Faculty of Epidemiology and Population Health, and Tuberculosis Centre, London School of Hygiene & Tropical Medicine, London, United Kingdom; 2 Institute of Epidemiology and Health, and Centre for Infectious Disease Epidemiology, Faculty of Population Health Sciences, University College London, London, United Kingdom; Liverpool School of Tropical Medicine, UNITED KINGDOM

## Abstract

**Background:**

Tuberculosis (TB) rates in England are among the highest in high-income countries. Poverty and historic and current immigration from high TB incidence parts of the world are two major drivers of tuberculosis in England. However, little has been done in recent years to examine socio-economic trends in TB rates in England, and to disentangle the role of deprivation from that of place of birth in the current TB epidemiology.

**Objectives:**

To assess the association between England’s 2008–2012 TB notification rates and small area-level deprivation, together and separately in the UK-born and foreign-born populations.

**Methods:**

Ecological analysis of the association between quintiles of England’s 2010 Index of Multiple Deprivation (IMD) and TB rates at the Lower-layer Super Output Area (LSOA; average population ~1500) level, using negative binomial and zero-inflated negative binomial regression models, adjusting for age, sex, urban/rural area classification, and area-level percentage of non-White residents.

**Results:**

There was a log-linear gradient between area-deprivation levels and TB rates, with overall TB rates in the most deprived quintile areas three times higher than the least deprived quintile after adjustment for age and sex (IRR = 3.35; 95%CI: 3.16 to 3.55). The association and gradient were stronger in the UK-born than the foreign-born population, with UK-born TB rates in the most deprived quintiles about two-and-a-half times higher than the least deprived quintile (IRR = 2.39; 95%CI: 2.19 to 2.61) after controlling for age, sex, urban/rural classification and percentage of non-White residents; whereas the comparable figure for foreign-born persons was 80% higher (IRR = 1.78; 95%CI: 1.66 to 1.91).

**Conclusions:**

Socio-economic deprivation continues to play a substantial role in sustaining the TB epidemic in England, especially in the UK-born population. This supports the case for further investigations of the underlying social- determinants of TB.

## Background

The tuberculosis (TB) annual notification rates in England remain among the highest in high-income countries, over four times higher than in the USA [1], and two to three times higher than in France, Germany and the Netherlands [2] for example. The greatest burden of disease is reported in people born outside of the United Kingdom, especially from high TB burden parts of the world [3]. The disproportionate burden of disease in these population groups is well established, and has received wide attention both in terms of investigation and public health policies [4–6]. A steady decline in the number of foreign-born TB cases in England has been reported in the past five years [7], possibly due to a combination of lower immigration from high burden countries (with annual TB rates greater than or equal to 150 per 100,000) and public health measures (e.g. 2012–2013 introduction of pre-entry TB screening for 101 countries with high TB incidence [8, 9]). Whilst the heightened attention to TB in foreign-born persons at higher risk is warranted, it has overshadowed the fact that the rapid decline in TB rates observed among UK-born persons up to the early 1980_s_ ceased from the mid-1980s and plateaued for over 20 years, until 2012 when they started to decline again [3].

In the UK-born population, TB seems to concentrate mainly in the most vulnerable and socio-economically deprived segments of the population, in which there is some evidence of ongoing local transmission [7, 10]. The failure of rates in the UK-born population of England to decline as fast as in foreign-born occurs in spite of the country being among the wealthiest in the world, and therefore able to implement the most effective TB control interventions and tools available to eliminate the disease as a public health problem. Reviews by the World Health Organisation (WHO) of their TB incidence projections suggest that in countries with low TB incidence (i.e. annual TB notifications <10 per 100,000 people), the full implementation of current TB control tools need to be supplemented by additional measures to meet the post-2015 global TB strategy goal of pre-elimination levels (annual notifications <1 per 100,000) by 2035 and elimination levels (annual notifications <1 per million) by 2050 [11]. These include increased efforts and actions to address and reduce the underlying social determinants of tuberculosis [11].

Social deprivation and poverty are among the oldest known drivers of TB, partly supported by the correlation between improvements in living and nutrition standards, and the rapid decline in TB in Western Europe during the first-half of the 20^th^ century [12], before the large-scale introduction of medical technologies like chest X-Ray screening, antibiotic therapy and vaccination [13]. Because of the resurgence of TB in England in the mid-1980s to early 1990s, several studies examined the contribution of social deprivation to reversing TB rates decline (Table 1). Using routine data, several ecological analyses measured the association between TB notification rates and area-level deprivation, consistently reporting higher TB rates in the most deprived areas of the country. While these studies helped raise awareness on the disproportionate TB burden in deprived communities, and prioritise resource allocation, most struggled to disentangle the role of deprivation from that of place of birth in explaining higher TB rates in deprived areas [14, 15] (Table 1). The population density of foreign-born persons, especially from low-income high-TB burden countries, is higher in metropolitan areas where there are more employment opportunities, with the greater proportion often residing in deprived areas where accommodation may be more affordable. Therefore, high TB rates in deprived areas could simply be explained by higher proportions of foreign-born residents at higher risk of TB, rather than social inequalities.

**Table 1:** Overview of previous ecological studies of the association between TB notification rates and area-level deprivation in England.

First Author	Year	Setting & Study Unit	Years of TB notifications	Deprivation Measure	Other measures	Analysis	Results	Limitations
Spence [28]	1993	33 Electoral Wards of Liverpool	Notifications 1985–1990	Townsend^†^ & Jarman^‡^	% free school meals & % council housing	Correlation coefficient	Strong correlation between deprivation and overall TB rates (association remain when ethnic minorities excluded)	No distinction UK born vs Non UK born
Bhatti [15]	1995	Local Authorities in England and Wales (403 in total in England & Wales) (and Hackney)	Notifications 1980–1992	Jarman Census 1991	None	Indirect age-sex standardisation using national rates	Proportion increase by ethnic groups. Increase in 30% poorest but not in remainder. Strong association with overcrowding	No distinction UK born vs Non UK born
Mangtani [32]	1995	32 London Borough of London	Notifications 1982–1991	Townsend and Carstairs^¥^	Unemployment overcrowding social class and proportion migrants	Indirect standardisation and Poisson regression	Proportion migrants and overcrowding associated with TB rates, but not associated with trends in TB rates	No distinction UK born vs Non UK born
Tocque [38]	1998	33 London boroughs and 36 metropolitan districts in England	Notifications 1991	Jarman		Correlation coefficient and Poisson regression	Positive correlation Jarman and TB rates, but less strong correlation when immigration component removed from Jarman index	No distinction UK born vs Non UK born
Hawker [33]	1999	39 Electoral Wards of Birmingham	Notifications 1989–1993	Townsend score	Ethnicity	Linear regression	Association overcrowding in White population but not Asians	No distinction UK born vs Non UK born
Tocque [34]	1999	Council wards in Liverpool	Average Annual TB rate 1981–85 and 1991–95	Jarman	Ethnicity	Multivariable regression	Positive association TB rates and unemployment in 1981, but in 1991, overcrowding, elderly living alone and proportion household with head from new commonwealth	No distinction UK born vs Non UK born
Bennett [26]	2001	Electoral Wards of Manchester, Liverpool, Birmingham and Cardiff	Hospital admissions 1991–1995	Jarman, Townsend and Carstairs	% born in India and Pakistan	Multilevel Poisson	Main explanatory is percentage born in India and Pakistan	No distinction UK born vs Non-UK born
					% residences overcrowded % residence / not owner occupied			
Parslow [27]	2001	Electoral Wards of Leeds	Cases aged 0–18 years in Leeds Chest clinic register 1982–1997	Carstairs	Proportion non-White children <19yrs and population density	Negative binomial regression of age sex standardised rates	TB rates associated with ethnicity but not deprivation; TB in non-ethnic minority associated with deprivation	No distinction UK born vs Non-UK born

^†^Townsend index [39] is an overall deprivation index of material deprivation based on 4 census indicators (% economically active residents aged>16 years, % households with no car, % owner occupied houses and % houses with >1 persons-per-room.

^¥^Carstairs index [40] is an indicator of material deprivation based on 4 census indicators (unemployment among men, car ownership, low social class and overcrowding).

^‡^Jarman index [41] is a composite measure of deprivation designed to identify underprivileged areas where social factors may be associated with higher General Practitioners workload, based on 8 census-derived variables (% old-age pensioners living alone, number of children <5 years, % single parent families, number of unemployed residents, number of unskilled workers, poor housing, overcrowding [% households with >1 person-per-room], population mobility [households who moved residence at least once a year], and % households headed by a person from ethnic minority or born in the new commonwealth).

The introduction of a central TB surveillance system in England in 1999, which systematically collects information on place of birth and place of residence of notified TB cases provided the opportunity to investigate the association between area-level deprivation and TB rates separately in UK-born and foreign-born persons, thus exploring the respective roles of area-level socioeconomic inequalities and place of birth. In this study, we estimated the overall association between the 5-year (2008 to 2012) overall tuberculosis rates and small area-level deprivation, as well as separately in UK-born and foreign-born populations. We in additi0n explored the relationship between TB in children aged 0 to 14 years (a proxy-measure for local TB transmission given most childhood cases likely result from recent infection) and area-level deprivation.

## Methods

### Study design, setting and study unit

This was an ecological study using TB notification rates in England from 2008 to 2012.

The unit of analysis was the Lower-layer Super Output Area (LSOA), which consist of the aggregation of several neighbouring Output Areas (OAs). OAs are small geographical units of fairly similar population size (between 40 and 250 residential households (average ~125), with estimated 100 to 625 residents each) and socially homogenous (including in terms of household tenure, dwelling type, and rural-urban status), defined and used by the UK Office for National Statistics (ONS) since 2001 to collect data and generate small-area statistics. By comparison, data collection in England was previously based on enumeration districts of various population size, whose shape and size were determined by data collection requirements rather than social homogeneity [16], thus providing less accurate socio-economic data and lower geographic resolution. LSOAs are obtained by grouping OAs in small areas roughly similar in population size (400 to 1200 households, 1000 to 3000 residents); this is the lowest level at which the data used by the ONS to generate area-level deprivation indices are aggregated [17]. England is divided by the ONS into 171,372 OAs, which are grouped into 32,844 LSOAs; all LSOAs were included in the analysis.

### Data sources

*TB Notifications* for 2008–12 were obtained from the Enhanced Tuberculosis Surveillance System (ETS) at Public Health England (PHE). We obtained anonymised information on age, sex, self-reported ethnicity, and UK-born status for each case. Individual residential address postcodes at the time of diagnosis were georeferenced to the corresponding LSOA using the ONS geographic codes database. The total number of cases by 5-year age groups, sex and place of birth was aggregated for each LSOA.

#### Population data

The ONS 2011 census data were used to obtain LSOA-level population statistics:

- The *resident population by place of birth* (overall and disaggregated in UK-born and foreign-born); these were used as denominator to obtain LSOA-level rates.
- The total *resident population by sex and 5-year age groups*;
- The total population by ethnic group (in ONS standard classification of self-reported ethnicity, then dichotomised into *White* (including White British, English, Irish, Scottish, Welsh, and any Other White) versus *non-White ethnicity* (ethnic group other than White, irrespective of country of birth);
- The sub-total of *foreign-born persons born out of the European Union* (EU) (henceforth labelled ‘*non-EU foreign-born’*). This variable was readily available from published small-area census statistics; given that England’s resident population born in EU countries other than the UK represents about a third of the foreign-born population [18], this variable (non-EU foreign-born) allowed us to distinguish them from the foreign-born population originating from any of the 27 other EU countries, none of which are considered by the WHO to be high-burden TB countries.

#### Area-level deprivation measures

We also obtained from the ONS the most recent (2010) version of England’s LSOA-level index of multiple deprivation (IMD)–a multidimensional measure of small-area socio-economic deprivation—and the domain specific deprivation indices ([i] income, [ii] employment, [iii] health and disability, [iv] education, skills and training, [v] barriers to housing and services, [vi] crime, and [vii] living environment; see [17] for details). These seven domains represent distinct but strongly correlated forms of deprivation that may be experienced by residents of an area. IMD scores are used to rank LSOAs across the country based on their relative deprivation levels, from most deprived (highest scores) to the least deprived (lowest scores) [17].

#### Other area-level characteristics

We also obtained for each LSOA its urban/rural classification from the ONS 2011 Rural-Urban Definition for Small Area Geographies. LSOAs are classified into broad categories taking into account population sparsity, respectively Urban (defined as connected built-up areas with 10,000 people or more, with sub-groups including [i] major conurbation, [ii] minor conurbation, and [iii] city and town), and Rural (less than 10,000 residents, including [iv] town and fringe, [v] village, and [vi] hamlets and dispersed/isolated dwellings); for details, see [19]). The information was regrouped into three levels, (i) major conurbation, (ii) minor conurbation, cities and towns, and (iii) rural areas.

### Outcome and exposure definitions

The primary outcomes were LSOA-level 5-year average annual TB notification rates respectively in all resident population, and separately in UK-born and foreign-born (non-UK born) persons. TB rates in children aged 0 to 14 years—a proxy-measure for local transmission given that childhood TB is most likely caused by recent transmission [20]–were also computed for subgroup analyses. LSOA TB notifications were used as numerators, and the respective population data as denominators. Robust standard errors were used to compute the 95% confidence intervals (95%CI) while accounting for area-level clustering. Age was available for all cases, but about 5% had missing information on whether they were UK-born or not, and they were not included in the analyses stratified by place of birth.

The main exposure was quintiles of (small) LSOA-level index of multiple deprivation (IMD) rank.

### Statistical analysis

The association between exposure variables and LSOA-level TB rates was measured using count-data regression models [21], with the log-transformed 5-year population denominator as the offset.

For each main analysis, the most appropriate count-data regression model was determined by comparing a standard Poisson model to the Negative Binomial model in order to mitigate the likely overdispersion of TB rates between LSOAs. We computed the mean number of events per LSOA and the variance to assess deviation from the key Poisson regression assumption that the conditional variance of the dependent variable is equal to the conditional mean [21, 22]. We also fitted both the Poisson and Negative Binomial regressions to the data and compared models’ fitness using the log-likelihood based Akaike’s Information Criterion (AIC) and Bayesian Information Criterion (BIC). The model with the best fit to the data (smaller AIC and BIC) was retained for final analyses [21, 22].

Many areas have small to no foreign-born population, therefore no foreign-born TB cases [7]. To address the zero counts, we used the Vuong Test [23] to further compare the Poisson and Negative Binomial models to their respective zero-inflated counterpart, using the non-EU foreign-born population as the predictor for ‘excess’ zero counts.

The final analyses for overall and foreign-born TB rates were carried out using zero-inflated negative binomial regression models, with non-EU foreign-born population used as the excess zero counts’ predictor, and the UK-born TB rates were analysed using a standard negative binomial regression model. All 95% confidence intervals (95%CI) were obtained using robust standard errors.

To measure the association between quintiles of area-level deprivation and LSOA-level TB notification rates, we first computed crude incidence rate-ratios (IRR), then age-and-sex adjusted RRs. We adjusted for confounding by age and sex by including in the model parameters their joint distribution within each LSOA (i.e. male-aged 0–14 years, female-aged 0–14 years, male-aged 15–64 years, female-aged 15–64 years, male aged over 64 years, and female aged over 64 years), as suggested by Morgenstern, to minimise the risk of ecological bias due to confounders’ misspecification [24]. Finally, we fitted a final multivariable model that measured the effect of area-level deprivation while further adjusting for LSOAs proportion of non-White population, as well as their urban/rural classifications. A sub-analysis of the association between area-level quintiles of deprivation and TB rates in children aged 0–14 years was also performed. All statistical analyses were done using Stata 14^®^ (StataCorp); significance testing was done using the Wald test.

### Ethical considerations

This study used anonymous data from routine surveillance obtained from Public Health England, and publicly available data from the Office for National Statistics.

## Results

About 40,000 TB cases were notified in England between 2008 and 2012, corresponding to a 5-year average annual notification rate of 15/100,000 persons. There was no apparent trend in annual rates over that period. The average annual notification rate in UK-born persons was 4.5/100,000 (95%CI 4.3 to 4.7 per 100,000), while the average rate in the foreign-born population was 80/100,000 persons (95%CI 78.5 to 82.6 per 100,000). The distribution of cases and corresponding average annual rates by LSOA characteristics is presented in Table 2. Overall, 71% of TB cases in England over the study period were reported from the most deprived two-fifth areas, with this trend similar in UK-born and foreign-born populations. However, there were differences between these two groups regarding other LSOA characteristics. Major conurbations accounted for 56% of UK-born TB cases compared to 70% for foreign-born cases. Nearly half UK-born TB cases were reported from areas with <20% non-White population, whereas 60% of foreign-born TB cases occurred in areas with ≥40% non-White residents. This distribution of cases was similar in relation to the LSOA percentage of non-EU foreign-born residents. There was a strong correlation between LSOAs’ percentages of non-White and percentage of non-EU foreign-born residents (Pearson’s correlation coefficient = 0.94; p<0.001), so we used the former as a covariate in subsequent analyses.

**Table 2:** Tuberculosis cases distribution and 5-year average annual notification rates by LSOA characteristics in England 2008–12.

	UK born			Foreign-born			All TB cases		
	Number of TB cases (column %; n = 10184)	population at risk	5-year average annual rate per 100,000 (95%CI)	Number of TB cases (column %; n = 29524)	population at risk	5-year average annual rate per 100,000 (95%CI)	Number of TB cases (column %; n = 39708)	population at risk	5-year average annual rate per 100,000 (95%CI)
**LSOA Quintiles of Index of Multiple Deprivation**									
Most deprived	4055 (40)	8574432	9.5 (9.1;9.9)	13053 (44)	2199206	119 (116.0;122.0)	17108 (43)	10773638	31.8 (30.7;32.8)
2	2431 (24)	8818466	5.5 (5.3;5.8)	8602 (29)	1861055	92.4 (88.9;96.1)	11033 (28)	10679521	20.7 (19.8;21.6)
3	1617 (16)	9255810	3.5 (3.3;3.7)	4271 (14)	1349267	63.3 (60.5;66.3)	5888 (15)	10605077	11.1 (10.6;11.6)
4	1173 (12)	9488578	2.5 (2.3;2.6)	2142 (7)	1018227	42.1 (39.4;44.9)	3315 (8)	10506805	6.3 (6.0;6. 7)
Least deprived	908 (9)	9538031	1.9 (1.8;2.0)	1456 (5)	909384	32.0 (30.2;33.9)	2364 (6)	10447415	4.5 (4.3;4.8)
**Rural / Urban classification of LSOA**									
Rural	781 (8)	8642493	1.8 (1.7;1.9)	447 (2)	472892	18.9 (17.0;21.0)	1228 (3)	9115385	2.7 (2.6;2.9)
Cities/minor conurbations	3707 (36)	22467460	3.3 (3.2;3.4)	8359 (28)	2580276	64.8 (62.7;67.0)	12066 (30)	25047736	9.6 (9.3;10.0)
Major conurbations	5696 (56)	14565364	7.8 (7.6;8.1)	20718 (70)	4283971	96.7 (94.4;99.1)	26414 (67)	18849335	28.0 (27.3;28.8)
**Percentage of non-White residents in LSOA**									
0–19.9%	4985 (49)	37612660	2.7 (2.6;2.7)	5815 (20)	3036658	38.4 (37.2;39.4)	10800 (27)	40649318	5.31 (5.29;5.4)
20–39.9%	1894 (19)	4480422	8.5 (8.0;8.9)	5851 (20)	1653640	70.8 (68.5;73.1)	7745 (20)	6134062	25.3 (24.5;26.0)
≥40%	3305 (32)	3582235	18.5 (17.7;19.3)	17858 (60)	2646841	135 (132.0;138.0)	21163 (53)	6229076	67.9 (66.2;69.7)

The variance of TB case count overall and in foreign-born person at the LSOA-level was 5 times higher than the mean, consistent with large overdispersion and excess zero counts. When we compared regression models for the association between TB rates and area-level deprivation, the zero-inflation negative binomial regressions provided better fits for overall TB rates and TB rates in foreign-born people, compared to Poisson regression models. For both outcomes, and for both the Poisson model and the negative binomial regression models, the Vuong tests comparing the standard version to the zero-inflation version of the regression models found p<0.001; and AIC and BIC for negative binomial regression models were lower than for Poisson regression model (Table 3)). These suggested overall that the zero-inflated negative binomial regression model was more appropriate to analyse TB rates overall and in foreign-born persons at the LSOA-level.

**Table 3:** Comparison of four count-data regression models of the association between area-level deprivation and TB notification rates.

	Poisson	Zero-inflated Poisson	Negative Binomial	Zero-inflated Negative Binomial
**All England population (Mean number TB case by LSOA = 1.21; Variance = 6.51)**				
Least Deprived	1	1	1	1
2	1.39 (1.30;1.50)	1.40 (1.30;1.51)	1.39 (1.29;1.49)	1.39 (1.30;1.49)
3	2.45 (2.29;2.63)	2.29 (2.14;2.44)	2.41 (2.26;2.58)	2.29 (2.15;2.44)
4	4.57 (4.28;4.87)	3.60 (3.39;3.83)	4.39 (4.12;4.68)	3.74 (3.52;3.98)
Most Deprived	7.02 (6.62;7.44)	4.82 (4.56;5.09)	6.66 (6.28;7.05)	5.22 (4.94;5.51)
p-value	<0.001	<0.001	<0.001	<0.001
AIC	113707.5	92882.9	88446.2	83753.0
BIC	113749.5	92941.7	88496.6	83820.2
Vuong test	<0.001		<0.001	
**Foreign-Born population (Mean number TB case by LSOA = 0.9; Variance = 4.98)**				
Least Deprived	1	1	1	1
2	1.31 (1.20;1.43)	1.20 (1.10;1.31)	1.27 (1.17;1.37)	1.19 (1.10;1.29)
3	1.98 (1.84;2.13)	1.64 (1.52;1.76)	1.83 (1.71;1.96)	1.62 (1.51;1.74)
4	2.89 (2.69;3.10)	2.19 (2.04;2.35)	2.58 (2.41;2.75)	2.15 (2.02;2.30)
Most Deprived	3.71 (3.48;3.95)	2.65 (2.48;2.84)	3.41 (3.20;3.62)	2.78 (2.61;2.96)
p-value	<0.001	<0.001	<0.001	<0.001
AIC	62010.2	59288.4	58147.3	56986.8
BIC	62052.2	59347.2	58197.7	57054.1
Vuong test	<0.001		<0.001	
**UK-Born population (Mean number TB case by LSOA = 0.31; Variance = 0.49)**				
Least Deprived	1		1	
2	1.30 (1.19;1.42)		1.30 (1.20;1.43)	
3	1.84 (1.68;2.01)		1.86 (1.84;2.13)	
4	2.90 (2.66;3.15)		3.01 (2.69;3.10)	
Most Deprived	4.97 (4.58;5.38)		5.19 (3.48;3.95)	
p-value	<0.001		<0.001	
AIC	47865.4		45865.0	
BIC	47907.4		45915.4	

For UK-born cases, the variance was only slightly higher than the mean, so the overdispersion was less severe; but the AIC and BIC suggested that the Negative Binomial was marginally better than the Poisson model to analyse the data, thus the negative binomial regression model was used to subsequently analyse TB rates in UK-born persons (Table 3).

The Overall TB rates increased with area-level deprivation, with crude rates in the most deprived quintile areas of the country more than five times higher than the least deprived quintile (IRR = 5.22; 95%CI (4.94 to 5.51)). But this association seemed partly explained by variation in the LSOAs age-and-sex composition, dropping to three times higher TB rates in the most deprived quintile after adjustment for age-and-sex (IRR = 3.35; 95%CI (3.16 to 3.55)) (Table 4). The log-linear gradient in overall TB rates by deprivation quintile was still present, albeit much weaker once controlling additionally for rural/urban area classification and the percentage of non-White resident in the area. The fully adjusted TB rates varied from 14% increase in the 2^nd^ least deprived to 81% higher in the most deprived quintile areas, when compared to the least deprived quintile of the country. The same multivariable model, controlling simultaneously for age, sex, deprivation, urban/rural classification and percentage of non-White residents, suggested that the overall TB rates were on average twice as high in urban than rural areas, and nearly six times higher in areas with ≥40% non-White residents compared to areas with <20% non-White residents (IRR = 5.78, 95%CI: 5.49 to 6.09).

**Table 4:** Association between LSOA-level deprivation and non-White population and TB notification rates in England in 2008–12.

	Crude			Age and Sex adjusted			Fully Adjusted^†^		
	IRR	95%CI	p-value	IRR	95%CI	p-value	IRR	95%CI	p-value
**Overall population**									
**Quintiles of LSOA Index of Multiple Deprivation rank**									
Least Deprived	1			1			1		
2	1.39	(1.30;1.49)		1.31	(1.22;1.41)		1.14	(1.07;1.21)	
3	2.29	(2.15;2.44)	<0.001^‡^	1.84	(1.73;1.96)	<0.001 ^‡^	1.35	(1.27;1.43)	<0.001^‡^
4	3.74	(3.52;3.98)		2.58	(2.43;2.74)		1.58	(1.50;1.67)	
Most Deprived	5.22	(4.94;5.51)		3.35	(3.16;3.55)		1.81	(1.71;1.91)	
**Percentage of non-White residents in LSOA**									
0–19.9%	1			1			1		
20–39.9%	3.31	(3.16;3.47)	<0.001	2.89	(2.75;3.04)	<0.001	2.60	(2.47;2.73)	<0.001
≥40%	8.7	(8.30;9.12)		6.96	(6.63;7.31)		5.78	(5.49;6.09)	
**Rural / Urban classification of LSOA**									
Rural	1			1			1		
Cities/minor conurbations	3.15	(2.95;3.37)	<0.001	2.01	(1.88;2.16)	<0.001	1.79	(1.67;1.91)	<0.001
Major conurbations	6.86	(6.43;7.32)		3.76	(3.50;4.03)		1.92	(1.78;2.06)	
**Foreign-born population**									
**Quintiles of LSOA Index of Multiple Deprivation rank**									
Least Deprived	1			1			1		
2	1.19	(1.10;1.29)		1.21	(1.12;1.31)		1.13	(1.05;1.23)	
3	1.62	(1.51;1.74)	<0.001^‡^	1.57	(1.47;1.69)	<0.001^‡^	1.34	(1.25;1.44)	<0.001 ^‡^
4	2.15	(2.02;2.30)		1.98	(1.85;2.12)		1.57	(1.47;1.68)	
Most Deprived	2.78	(2.61;2.96)		2.38	(2.22;2.54)		1.78	(1.66;1.91)	
**Percentage of non-White residents in LSOA**									
0–19.9%	1			1			1		
20–39.9%	1.72	(1.62;1.82)	<0.001	1.63	(1.54;1.73)	<0.001	1.49	(1.41;1.58)	<0.001
≥40%	3.10	(2.94;3.28)		2.64	(2.49;2.79)		2.28	(2.15;2.42)	
**Rural / Urban classification of LSOA**									
Rural	1			1			1		
Cities/minor conurbations	2.56	(2.29;2.85)	<0.001	2.17	(1.94;2.43)	<0.001	1.96	(1.75;2.19)	<0.001
Major conurbations	3.13	(2.79;3.50)		2.56	(2.28;2.87)		1.83	(1.63;2.05)	
**UK-born population**									
**Quintiles of LSOA Index of Multiple Deprivation rank**									
Least Deprived	1			1			1		
2	1.30	(1.19;1.43)		1.30	(1.18;1.42)		1.21	(1.11;1.33)	
3	1.86	(1.70;2.04)	<0.001^‡^	1.72	(1.58;1.89)	<0.001^‡^	1.45	(1.33;1.58)	<0.001 ^‡^
4	3.01	(2.76;3.28)		2.48	(2.27;2.70)		1.77	(1.63;1.93)	
Most Deprived	5.19	(4.78;5.63)		3.90	(3.59;4.24)		2.39	(2.19;2.61)	
**Percentage of non-White residents in LSOA**									
0–19.9%	1			1			1		
20–39.9%	3.20	(3.01;3.40)	<0.001	2.9	(2.71;3.10)	<0.001	2.39	(2.24;2.56)	<0.001
≥40%	6.99	(6.62;7.38)		5.96	(5.60;6.34)		4.25	(3.96;4.55)	
**Rural / Urban classification of LSOA**									
Rural	1			1			1		
Cities/minor conurbations	1.85	(1.70;2.01)	<0.001	1.44	(1.32;1.57)	<0.001	1.31	(1.20;1.42)	<0.001
Major conurbations	4.52	(4.17;4.91)		3.12	(2.86;3.4)		1.63	(1.49;1.79)	

^†^ Fully adjusted regression models include quintiles of area-level deprivation rank, and further adjusted for age, sex, urban/rural area classification, percentage of White/non-White residents.

^‡^ Test for trend.

There were, however, differences in the association between area-level deprivation and TB rates in UK-born versus foreign-born populations (see Table 4). The magnitude and gradient of association between deprivation and TB rates was much steeper in the UK-born population, in which the crude TB rates in the most deprived quintile areas were over five times higher than the least deprived quintiles (IRR = 5.19, 95%CI: 4.78 to 5.63), compared to only about three times higher in the foreign-born population (IRR = 2.78, 95%CI: 2.61 to 2.96). The difference in the association between area-level deprivation and TB rates in these two populations persisted after adjusting for age, sex, rural/urban classification and percentage of White/non-White residents. Compared to the least deprived quintile areas, the fully adjusted rate-ratios of association between area-level deprivation and TB rates in UK-born persons varied from 2.40 (95%CI: 2.19 to 2.61) in the most deprived quintile to 1.21 (95%CI: 1.11 to 1.33) in the 2^nd^ least deprived quintile areas; whereas equivalent figures in the foreign-born population were respectively 1.78 (95%CI: 1.66 to 1.91) and 1.13 (95%CI: 1.05 to 1.23) (Table 4).

Findings from the subgroup analysis of the association between area-level deprivation and TB rates in children aged 0–14 years (a proxy-measure for local TB transmission) are in Table 5. Overall, the pattern of association was similar to that among UK-born persons, with a strong gradient of higher TB rates with increasing deprivation levels; crude TB rates in children ≤14 years living in the most deprived quintile areas were 10 times higher than those in the least deprived areas (IRR = 10.07, 95%CI: 7.76 to 13.06). After adjusting for the urban/rural area classification and the percentage of non-White residents, there was still good evidence of association, albeit weaker, between area-level deprivation and TB rates in children ≤14 years old.

**Table 5:** Association between area-level deprivation and TB notification rates in children aged 0–14 years in England in 2008–12.

	TB notifications			Crude			Fully Adjusted^†^		
	TB cases	Population aged 0–14 years	Annual TB rate (per 100,000)	IRR	95%CI	p-value	IRR	95%CI	p-value
**Quintiles of LSOA Index of Multiple Deprivation rank**									
Least Deprived	77	1793732	0.86						
2	94	1698737	1.11	1.29	(0.92;1.80)		1.06	(0.76;1.49)	
3	220	1752179	2.51	2.89	(2.15;3.88)	<0.001^‡^	1.60	(1.19;2.16)	<0.001^‡^
4	520	1931600	5.38	6.14	(4.69;8.05)		2.23	(1.68;2.95)	
Most Deprived	1053	2308561	9.12	10.07	(7.76;13.06)		2.84	(2.14;3.78)	
**Percentage of non-White residents in LSOA**									
0–19.9%	443	6986089	1.27						
20–39.9%	372	1117089	6.66	5.27	(4.45;6.25)	<0.001	3.53	(2.95;4.23)	<0.001
≥40%	1149	1381631	16.60	13.05	(11.35;14.99)		7.10	(6.00;8.39)	
**Rural / Urban classification of LSOA**									
Rural	28	1470602	0.38						
Cities/minor conurbations	524	4437751	2.36	6.04	(3.96;9.20)	<0.001	2.85	(1.85;4.39)	<0.001
Major conurbations	1412	3576456	7.90	19.49	(12.9;29.44)		3.50	(2.27;5.41)	

^**†**^Adjusted for quintiles of LSOA IMD rank, percentage of non-White resident population and urban/rural area classification.

^‡^Test for trend.

## Discussion

Our analyses show that over the 5-year study period, English TB rates were positively associated with small-area level deprivation even after adjusting for age, sex, urban/rural differences and the area-level proportion of non-White residents. But the relationship between area-deprivation and TB rates was much stronger in the UK-born population compared to foreign-born; after controlling for confounders, TB rates in UK-born persons living in the most deprived quintile areas of the country were 2.4 times higher than the least deprived quintile areas (95%CI 2.19 to 2.61), compared to just about 80% higher rates in foreign-born persons for the same comparison. Area-level deprivation was also strongly associated with higher TB rates in children aged 0–14 years old.

An advantage of the current study over previous ecological analyses of the relation of deprivation to TB rates in England was the availability and use of LSOAs as unit of analysis, with the small populations providing better geographic resolution and more homogeneous population groups (for example England was formerly divided by the ONS in 9,265 census wards versus about 32,000 LSOAs currently); this also minimises ecological bias when measuring the association between area-level deprivation and the risk of TB [21, 25]. By comparison, previous ecological studies have used larger and more heterogeneous aggregation levels, ranging from electoral wards (population varying from 5000 to 26000 per wards) [26–28] to local authorities (35,000 to nearly 1,000,000 residents per unit) [15]. We also used exact census-derived population counts as denominators to calculate TB rates, which helped to avoid some pitfalls of population estimates, for example underestimating small groups like foreign-born populations.

A possible limitation of this study is TB cases undernotification; an audit of the TB surveillance system between 1999–2002 using the capture-recapture method estimated about 15% under-reporting, but improving with time [29]. This audit did not explore difference in under-reporting between UK-born and foreign-born populations, making it difficult to predict the likely impact of any under-notification on our findings. It is plausible that under-notification is more frequent in the UK-born population, helped by the under-notification of post-mortem TB diagnosis in elderly persons, and differential clinical suspicion index [30], although barriers to accessing healthcare (e.g. language, unfamiliarity with the system) may also affect case detection in foreign-born population. We note that under-notification of TB cases, whether not associated to area-level deprivation (i.e. similar in more and least deprived areas), or if more frequent in more deprived area, would lead to an underestimation rather than overestimation of the association of area-level deprivation and TB rates, which means that the estimates presented here are likely conservative. Another potential limitation of this study is the fact the TB notification data used cover the period of 2008 to 2012. Although the data is not recent, this provided us with a more accurate pairing of persons with TB (numerator data) to the LSOA denominator population (based on the most recent 2011 census) and area-level deprivation index (IMD most recently generated in 2010), thus allowing more accurate analysis. Furthermore, while TB rates have been declining in the UK since 2012, including in the UK-born population, surveillance data show that there is still substantial inequalities across deprivation levels, with for example rates of 16.6 per 100,000 in the 10% of the population living in the most deprived areas, compared to only 3.0 per 100,000 in the 10% living in the least deprived area in 2018 [3].

The respective roles of social deprivation and immigration from places with higher TB levels in explaining the resurgence of TB in England have been the object of much debate [14]. Most authors have argued that most of the disease resurgence is related to immigration from high-TB parts of the world, but some are also of the opinion that similar to comparable low-TB incidence settings, poverty and social deprivation have played an additional role in the failure of TB rates to decline since the mid-1980s [31]. It is true that the greater burden of disease in England nowadays rests with foreign-born populations. Our results suggest that deprivation is an important risk factors for TB in both UK-born and foreign-born population, but plays a greater role in differential TB rates in the UK-born population compared to the foreign-born population. In contrast Bennet *et al* reported that in their analyses of TB rates in electoral wards in Manchester, Liverpool, Birmingham and Cardiff, variations in rates were explained by the proportion of residents born in India and Pakistan, and not by area-level deprivation [26]. The difference to our results could be explained by the fact that these authors did not examine the association separately in UK-born and foreign-born persons, and their study was set only in major conurbations with high proportion of foreign-born persons. The weaker association between deprivation and TB rates in foreign-born populations in our study is most likely due to the fact the risk of disease is mostly associated with the higher TB burden in place of origin of many foreign-born persons in England, and their ties to these areas, with deprivation only playing a smaller role. We note however that while the overall association between TB and area-level deprivation is less strong in the foreign-born population, a greater proportion (>55%) lives in lowest two quintiles deprived areas.

On the other hand, we found a stronger association between deprivation and TB in UK-born populations, which is consistent with the well-known historical link between TB and poverty, with greater prevalence of deprivation-related TB risk factors in poorer areas. These may include living in overcrowded (and possibly poorly ventilated) dwellings, as well as poorer nutritional status, both associated with higher risk of TB infection as well as progression to active disease [20]. These results are consistent with findings from the previous studies reporting an association between higher TB rates and overcrowding in London boroughs [32], as well another where overcrowding was associated with TB rates in the White population in Birmingham, but not the Asian population [33]. Tocque *et al* also reported a positive association between TB rates in Liverpool council wards and unemployment, a known predictor of financial status and related circumstances (housing, nutritional status etc.) [34]. Furthermore, it was found in surveys that other health determinants associated with higher risk of TB, including for instance tobacco smoking, history of incarceration, and history of homelessness, are more prevalent in socially deprived population subgroups [35]. Finally, the strong association between area-level deprivation and overall TB rates in children aged 0–14 years in our study supports the hypothesis that deprivation remains an important determinant of recent TB transmission; most TB cases in children result from recent *Mycobacterium tuberculosis* infection [36], suggesting ongoing transmission in the most deprived areas. But this association is also probably explained in part by family ties to high TB-burden parts of the world.

## Conclusions

In summary, the results presented here are the first in over 15 years examining variations in TB rates across gradients of area-level deprivation, and the first since the introduction of the centralised TB surveillance system, as well as the current high-resolution measure of area-level deprivation. Despite their limitations, the findings suggest that deprivation continues to play a role in sustaining the TB epidemic in the UK-born population in England and deserve further attention. Further studies are warranted to investigate individual components of the IMD, and which deprivation-related determinants of health are related to the risk of TB at the individual-level. Such studies could be helpful in designing and planning interventions to address the social determinants of TB and help progress towards TB elimination in the near future. Our results should also contribute to raising the awareness of the disproportionate burden of TB in the UK-born populations residing in the most deprived areas. More generally, these findings highlights the stark health inequalities still present in one of the wealthiest countries in the world, and the importance of meaningful social interventions, including for example improved housing, food security and smoking cessation, Such structural interventions are likely not only to help further reduce TB incidence, but they will also have far reaching health benefits in the society [37].
